# Fluctuated water depth with high nutrient concentrations promote the invasiveness of *Wedelia trilobata* in Wetland

**DOI:** 10.1002/ece3.5941

**Published:** 2019-12-20

**Authors:** Jianfan Sun, Qaiser Javed, Ahmad Azeem, Ikram Ullah, Muhammad Saifullah, Rakhwe Kama, Daolin Du

**Affiliations:** ^1^ School of the Environment and Safety Engineering Jiangsu University Zhenjiang China; ^2^ Key Laboratory of Modern Agricultural Equipment and Technology Ministry of Education Institute of Agricultural Engineering Jiangsu University Zhenjiang China

**Keywords:** competitive interaction index, interaction, invasion, invasive species, nutrients, Water depth

## Abstract

The distribution of invasive and native species in wetlands is determined by hydrological conditions; whereas conditions such as water depth fluctuations, variations in the nutrient concentrations are expected to affect the growth and physiological traits of plants. For the assessment of such effects, we conduct greenhouse experiment with three factors; 1) water depth of 5 cm and 15 cm (static and fluctuated); 2) three levels of nutrient concentrations (i) full‐strength Hoagland solution (N1), (ii) ¼‐strength Hoagland solution (N2), and (iii) ^1^/_8_‐strength Hoagland solution (N3); and 3) species, invasive *Wedelia trilobata* (L.) and its congener, native *Wedelia chinensis* (Osbeck.) under mono and mixed culture. Water depth of 5 cm combined with any of the nutrient treatments significantly restrained the photosynthesis, intracellular CO_2_ concentration and leaf chlorophyll of both *W. trilobata* and *W. chinensis*. Increase in the water depth to 15 cm with low‐nutrient treatment N3 did not sustain the physiological traits of *W. chinensis* under mono and mixed planting. A great loss was noted in the growth of *W. chinensis* at 15 cm static and fluctuated water depth with low‐nutrient treatment (N3) and under mixed culture. In addition, water depth fluctuations with both low‐ and high‐nutrient treatments significantly affected the root‐shoot ratio, relative growth rate, and interspecific interaction among these two species. *W. trilobata* benefited more from competitive interaction index (CII) under fluctuated water depth at 15 cm with high nutrients, and the value of CII was clearly positive. Therefore, higher competitive ability may contribute to the invasiveness of *W. trilobata* in wetlands.

## Graphical abstract

In this work, the affect of water depth fluctuation with different nutrient treatments on the growth has been predicted for Wedelia trilobata. Furthermore, our work focused on an important element as competitive interaction index among invasive Wedelia trilobata and its native W. chinensis for prediction of invasiveness of Wedelia trilobata in wetland ecosystem.

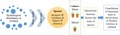

## 1 INTRODUCTION

Invasion by plant species poses a serious threat to native ecosystems and causes considerable losses to economy. Invasive plants develop their monocultures and dominate the invaded ecosystems by excluding the co‐occurring native plants. Accordingly, plant invasions alter structure of the native ecosystems in the wetlands. Numerous hypotheses have been projected to explain the success of invasions by the invasive plants (Daehler, 2003). Traits of invasive plant species have been investigated and compared in several studies in order to understand the concept of plant invasiveness (Van Kleunen, Dawson, Schlaepfer, Jeschke, & Fischer, 2010a; Skálová, Havlíčková, & Pyšek, 2012) with inconsistent results (Palacio‐López and Gianoli, 2011, Van Kleunen, Weber, & Fischer, 2010b). Godoy, Valladares, and Castro‐Díez (2011) compared twenty ecologically related phylogenetic species to observe performance of the invasive species and found that invasive species show higher performance under limited resources than native species. Further, based on the outcomes of these studies, high growth rate including physiology (Chen, Zhou, Yin, Liu, & Luo, 2013), leaf‐area, shoot distribution, size, and fitness (Kleunen, Weber, et al., 2010b) were noted for invasive species, due to their reproductive capacity (Willis, Memmott, & Forrester, 2000), high resource‐use efficiency (Radford, Dickinson, & Lord, 2007), fecundity (Ozaslan et al., 2016), and high phenotypic plasticity (Keser et al., 2015). Therefore, plant invasiveness may be determined under stressful conditions by considering some influencing key factors, such as physiology, biomass allocation (Godoy et al., 2011), and plant‐plant interactions (Wang, Zhang, Xu, & Yu, 2016a; Zhou et al., 2018).

Waterlogging or change in the water depth resulting from hydrological turbulences is a common stress for plants in wetland ecosystems (McGOWAN et al., 2011; Yuan, Yang, Liu, & Wang, 2017). The climate change is a strong reason for the hydrological disturbances such as tidal variation, flooding, and severe drought. It inevitably imposes stress on plant communities and affects the performance of plants in various transitional terrestrial and amphibious ecosystems (Wang & Li, 2016; Wright, Hornbach, McHugh, & Mann, 2015). Stress gradient hypothesis supported these studies, which used to predict the competition effect among the neighboring plants along the gradients of environmental or hydrological disturbance (Lortie & Callaway, 2006; Smit, Rietkerk, & Wassen, 2009; Yue et al., 2019).

In wetland ecosystem, it is difficult to maintain water depth at constant level because of fluctuations in water levels. However, hydrological disturbances such as variations in the water depth change the nutrients and affect the interspecific interactions among plants. The increased water depth increases the loss of nutrients and damage the plant tissues (Sasikala, Tanaka, & Jinadasa, 2008). change in water depth and nutrient levels mainly affect the net photosynthetic rate (*PN*), stomal conductance (gs), chlorophyll (Chl), transpiration rate (E), carbohydrate availability, and growth rate in many wetland plants (Colmer & Voesenek, 2009; Zhou et al., 2018). There are also some conflicting views about the waterlogging‐tolerant plants that can maintain their photosynthetic activity better, conserve energy and generate new tissues after reemergence of flooding (Bailey‐Serres & Voesenek, 2008; Nielsen, Podnar, Watts, & Wilson, 2013). Chen et al. (2013) found that invasive *Alternanthera philoxeroides* was more tolerant to fluctuating water depth than their native, congeneric *Alternanthera sessilis* in wetland and showed better photosynthetic capacity. Similar results were noted by (Xiao et al., 2010) for *Spartina alterniflora* in the coastal wetlands of China. Still, it is not clear whether the invasive plants have higher values of traits linked to photosynthetic activity including *PN*, gs, Chl, and growth properties, that is, root‐shoot fresh and dry weight, relative growth rate (RGR), and root‐shoot ratio (RSR) over native plants under different water depths and nutrient concentrations? Consequently, more research is required regarding the behavior of invasive plants and their congener native plants in wetlands for their better management.

Therefore, we have selected the world's most noxious invasive species *Wedelia trilobata (L.)* Hitchc*.* (Asteraceae) and its congener, native *Wedelia chinensis* (Osbeck.) Merr*.* for this study to contribute to the research area related to invasive plant behavior in wetlands. *W. trilobata* is native to tropical region of south America (Wang et al., 2012; Weber, Sun, & Li, 2008) and is a clonal evergreen creeping herb which was also found in southern region of China in 1970s on a large scale (Chen et al., 2019; Dai, Fu, Wan, et al., 2016b; Qi et al., 2014). In China, initially it was introduced as a groundcover species but later it spread rapidly from garden to roadsides and then to the agricultural fields (Song, Chow, Sun, Li, & Peng, 2010; Talukdar & Talukdar, 2013). While, *W. chinensis* the native congener of *W. trilobata* is mostly used as medicinal plant. The growth rate of *W. chinensis* is very slow as compared to *W. trilobata* (Dai, Fu, Qi, et al., 2016a; Si et al., 2013). According to results of previous studies related to invasion of *W. trilobata* in different environments, it is hypothesized that *W. trilobata* invade successfully in highly fluctuating water depth with high nutrient concentrations. Therefore, we selected *W. trilobata* and its native congener *W. chinensis* to examine (1) whether the water depth and nutrient fluctuations affect the growth and photosynthetic capacity of invasive species *W. trilobata* more or less than its native congener *W. chinensis* in mono and mixed culture? and (2) how the interspecific interactions among these species are affected by water depth and nutrient fluctuation in mixed culture? Results of this study will be helpful in the management of invasive species in wetlands based on competitive interaction, growth, and physiological responses.

## 2 MATERIALS AND METHODS

### 2.1 Site and plant material

The ramets of *W. trilobata* and *W. chinensis* were collected from the single location of Jiangsu University (32.20°N, 119.45°E), Zhenjiang, Jiangsu, P. R. China in early March 2019. For each species, 450 ramets were collected and then cultured in a sand medium. These were watered daily with tap water in order to adapt them to the greenhouse conditions (located at Jiangsu University, Zhenjiang, China). Seven‐days later, 360 ramets of each species with strong vivacity were selected for the following experiment. Before transplanting of the ramets, the average ramet height and weight were recorded which were 11.21 cm and 1.77 g, respectively, for *W. chinensis,* and 13.76 cm and 2.46 g, respectively, for *W. trilobata*. The plants were divided into two groups as monoculture and mixed culture. In monoculture, two‐ramets of each species per pot, while in mixed culture total two‐ramets of *W. trilobata* and *W. chinensis* were planted (one ramet for each species) in an internal pot (17.7 cm diameter × 12 cm height). These pots were filled with clean washed sand and then placed to outer pots of 28 cm diameter and 35 cm height.

### 2.2 Experiment design

Plants of both species were planted in the last week of April 2019 and were subjected to three nutrient concentration levels of Hoagland solution crossed with four different water depths in order to simulate the naturally occurring fluctuations of water in wetlands. For water depth fluctuations, four treatment levels were established: (1) static‐water level at 5 cm, coded as 5S; (2) fluctuated‐water level at 5 cm, fluctuating between 5 and 10 cm water depth, coded as 5F; (3) static‐water level at 15 cm, coded as 15S; and (4) fluctuated‐water level at 15 cm, fluctuating between 10 and 20 cm water depth, coded as 15F. The nutrient concentrations were applied as full‐strength Hoagland solution (N1), ¼‐strength Hoagland solution (N2), and ^1^/_8_‐strength Hoagland solution (N3). Five replicates were set up for each treatment, and total plastic pots were 180, 45 for each group (established water depth levels (1) × nutrient concentration levels (3) × species (3) × replicates (5)). Tap water was added daily to keep the water level maintained at static level of 5 cm and 15 cm for a week to ensure the plant survival during whole period of the experiment. Afterward, in first week of May 2019, water level maintained static at mentioned static levels and fluctuated up to mentioned fluctuation levels, and the nutrients solution was rehabilitated twice a week. Plants were harvested on July 2, 2019, that is, after eight weeks of the treatment.

## 3 MEASUREMENTS

### 3.1 Growth parameters measurements

Five plants were selected from each treatment for the measurement of growth parameters. The measurements chosen for growth trait analysis were as follows: FWS: fresh weight of shoot; DWS: dry weight of shoot; FWR: fresh weight of root; DWR: dry weight of root; TFW: total fresh weight of a plant; and TDBm: total dry biomass of a plant. FWS, DWS, FWS, and DWR were measured by using weighing scale.

### 3.2 Relative growth rate

The relative growth rate (RGR) within the species under treatments was calculated by following (Pérez‐Harguindeguy et al., 2013).

(1) $$Relativegrowthrate\left(\mathrm{RGR}\right)=\frac{\ln Ws2-\ln Ws1}{Ts2-Ts1}$$

Here; Ws2 is the weight of seedling at harvest, Ws1 is the weight of seedling at start the of stress treatments, Ts2 is harvest time and Ts1 is time of start of the experiment.

### 3.3 Root‐shoot ratios

It was computed by the following equation;

(2) RSR=DWR/DWS

where RSR is the root‐shoot ratio, DWR is the dry weight of root, and DWS is the dry weight of shoot.

### 3.4 Competitive interaction index

Competitive interaction index (CII) responses of both invasive *W. trilobata* and its native *W. chinensis* under mono and mixed culture were calculated based on the total dry weight of a plant. The CII is appropriate for evaluating interactions among two species either positive or negative. Through CII, the performance of each species can be compared when grown in mixed culture to its performance in monoculture. The CII was calculated by using the following equations (Armas, Ordiales, & Pugnaire, 2004; Liu, Yang, & Zhu, 2018):

(3) $$CIIx=\left(Axy-Ax\right)/\left(Axy+Ax\right)$$

(4) $$CIIy=\left(Ayx-Ay\right)/\left(Ayx+Ay\right)$$

where A is the total dry weight of a plant, while x and y represent to both the species separately, Ax shows the total dry weight of species x (invasive *W. trilobata*) when grown alone, and Ay is the total dry weight of species y (native *W. chinensis*) when is grown alone. Axy is the total dry weight of species x when grown with species y, and Ayx is the total dry of species y when is grown with species x.

### 3.5 Physiological parameters

Physiological parameters selected for measurements were as follows: *PN*: net photosynthetic rate; g_S_: stomatal conductance; and Ci: intercellular CO_2_ concentration. *PN*, g_S_, and Ci were measured by using a portable LI‐6400XT, *Lincoln*, USA photosynthesis measurement system. All these data were recorded during full‐sunshine at 9:30–11:30 a.m. once a week during treatments. The following settings were noted during data collection: Photosynthetic active radiation (PAR) was 800 μmol/m^2^ s^−1^, temperature 28°C and CO_2_ concentration were 500 μmol/mol. Leaves were selected from five plants per group of treatment for the measurements.

### 3.6 Leaf chlorophyll measurement

Plant chlorophyll meter, Oakoch OK‐Y104, made in China, was used to measure the leaf chlorophyll (Chl) contents (SPAD). The Chl was noted from the same leaves which were used for photosynthetic measurements.

### 3.7 Statistical analysis

All measurements were examined statistically through SPSS 17 software (SPSS Inc., IL, USA). Three‐way analysis of variance (ANOVA) was used for the data analysis to discriminate the effects of species, water depth fluctuations, different nutrient treatments and their interaction on physiological characteristics and plant growth properties under mono and mixed culture. The Tukey test was applied to determine the differences at 5% significance level (*p* ≤ .05) between means (*n* = 5).

## 4 RESULTS

### 4.1 Biomass, biomass allocation, and growth traits

*W. trilobata* and *W. chinensis* responded differently to different nutrient concentrations and water depth fluctuations under mono and mixed culture. In this study, based on ANOVA results (Table 1), both under the mono and mixed culture, FWS, DWS, FWR, and DWR were significantly affected by water depth, nutrient levels and the interaction between them (WD × N × S). The differences were significant among water depth and nutrient concentrations (*F* = 42.09, *p* < .001), among water depth and species (*F* = 47.82, *p* < .001), among nutrients and species (*F* = 16.45, *p* < .001), and interaction between all of them (*F* = 35.64, *p* < .001), respectively, for TFW of both species. Consequently, TDBm of both species were also significant in the interaction between water depth, nutrients, and species (*F* = 50.33, *p* < .001). Therefore, it was reflected from the results that water depth and nutrient levels affected the TFW and TDBm of *W. trilobata* at 15 cm static water depth with N2 and N3 nutrient concentrations under mixed culture, but it did not affect TFW and TDBm at 5 cm static and fluctuated water depth as compared to *W. chinensis* under monoculture as well as in mixed culture (Table 1).

**Table 1:** Three‐way ANOVA results with interactions among water depth, nutrients levels, and species for the effects of water depth fluctuations with different nutrients treatments on growth‐related data for *W. trilobata* and *W. chinensis*

Factors	Growth characteristics							
	FWS	DWS	TFW	FWR	DWR	TDBm	RSR	RGR
	(g)	(g)	(g)	(g)	(g)	(g)	−	(g/g day^−1^)
WD	1609.78**	2,515.60**	6,020.37**	456.54**	568.48**	9,574.25**	67.22**	5,449.52**
*N*	1,151.80**	5,254.70**	1911.80**	923.92**	882.58**	2,307.00**	65.43**	5,021.56**
S	956.73**	2,652.89**	9,402.68**	986.90**	492.91**	9,864.25**	38.22**	4,714.99**
WD* *N*	7.84**	17.60**	42.09**	2.983^ns^	10.05**	1832.00**	2.10^ns^	34.58**
WD*S	42.86**	16.52**	47.82**	27.489**	15.01**	432.58**	3.39*	194.06**
*N* *S	2.58**	13.68**	16.45**	4.412*	9.57**	341.00**	2.35^ns^	47.00**
WD* *N* *S	2.56**	33.95**	35.64**	2.759*	3.43**	50.33**	0.68^ns^	29.68**

Abbreviations: *N*, nutrient treatments; ns, nonsignificant; S, species; WD, water depth.

* indicates significant values at *p* < .005.

** indicates significant values at *p* < .001.

**Figure 1:**
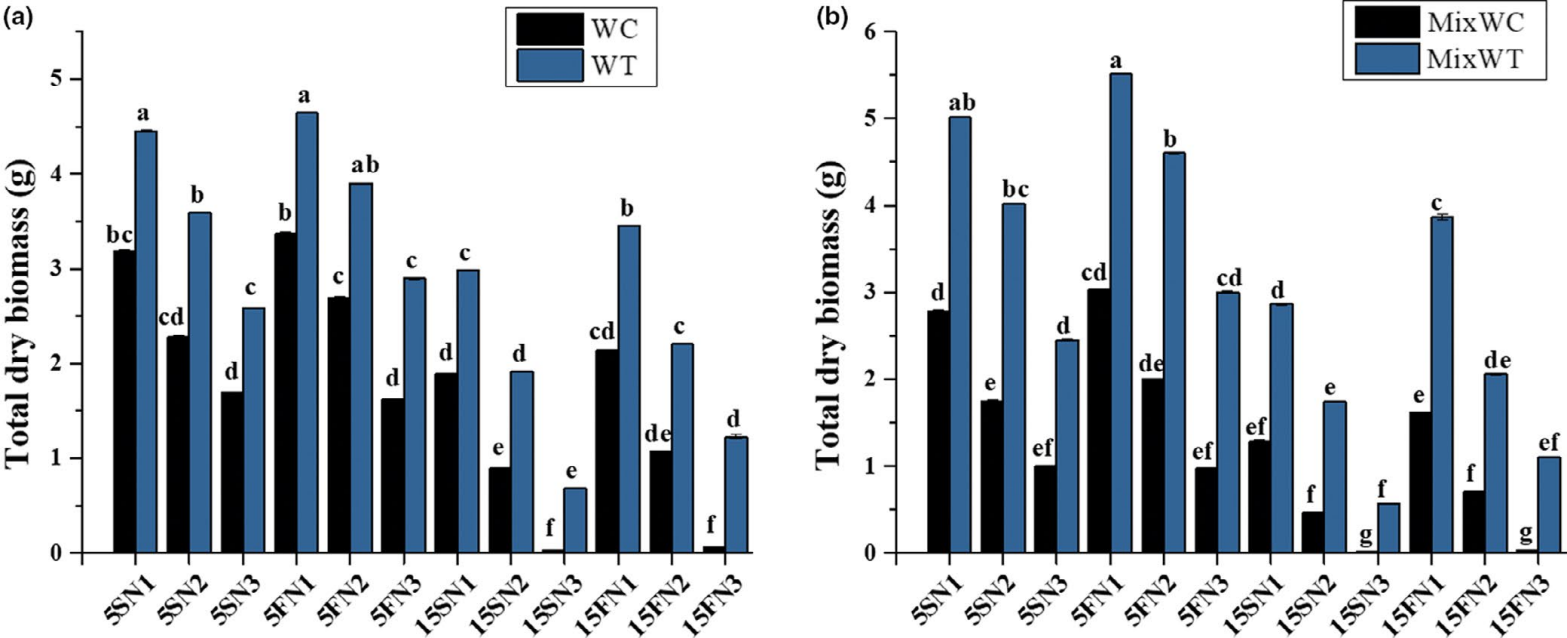
Effects of water depth fluctuations with different nutrient treatments on the (a) total dry biomass in monoculture, (b) total dry biomass in mixed culture of *W. trilobata and W. chinensis*. Mean + SE with different letters indicate a significant difference among mono and mixed culture treatments (at *p* < .05)

The TDBm was higher at 5FN1 for both the species under mono and mixed culture. In the mono and mixed culture, TDBm of native *W. chinensis* was decreased by increasing water depth to 15 cm and decreasing the nutrients concentration (N2 and N3) (Figure 1a and b). However, *W. trilobata* had higher TDBmat 5 cm static and fluctuated water depth (5S and 5F) with all levels of nutrient concentrations (N1, N2, and N3) in monoculture. Followed by mixed culture at 15 cm fluctuated water depth (15F) with nutrient concentrations of N1 and N2, *W. trilobata* also showed higher TDBmthan *W. chinensis*. Moreover, biomass as DWS, DWR, and TDBm of a plant for both species were also affected by water depth and nutrients fluctuations (Table 1; Figure 1).

**Figure 2:**
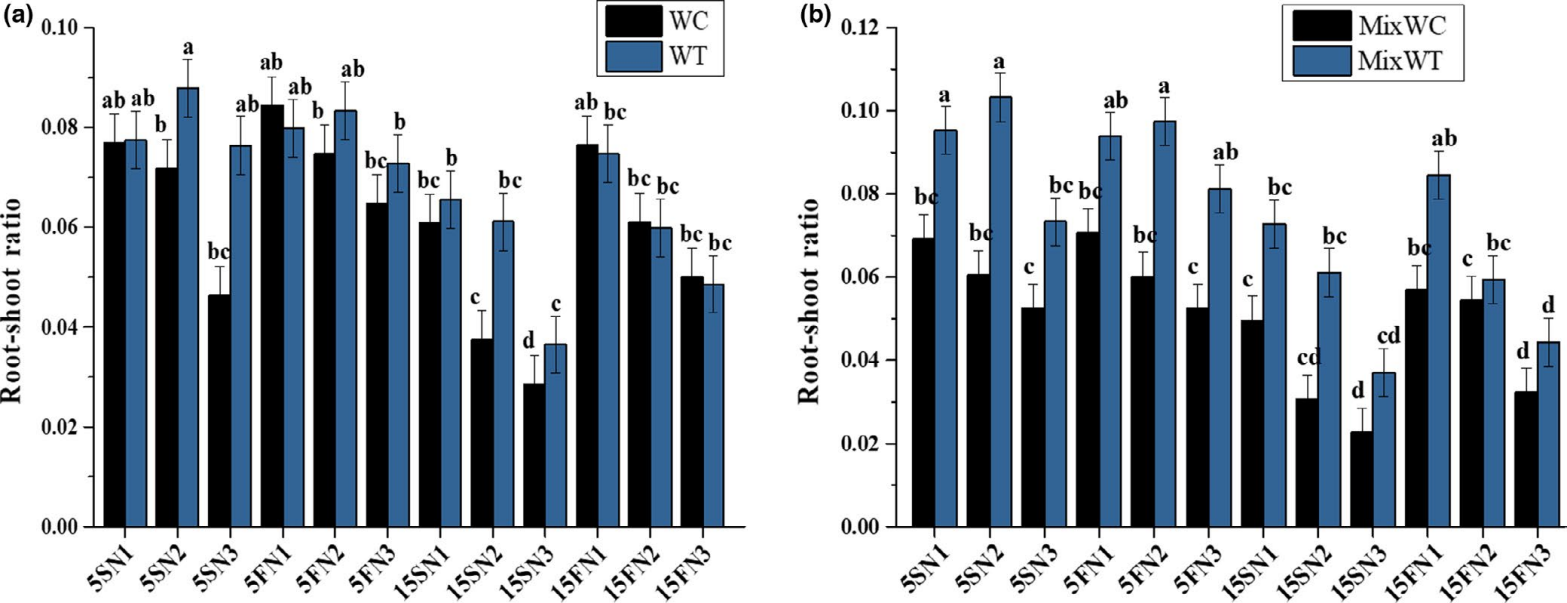
Effects of water depth fluctuations with different nutrient treatments on the (a) root shoot in monoculture, (b) root‐shoot ratio in mixed culture of *W. trilobata and W. chinensis*. Mean + SE with different letters indicate a significant difference among mono and mixed culture treatments (at *p* < .05)

The tested plants showed a significant difference in growth traits like as RSR under both mono and mixed planting. Significant difference was recorded between water depth and species (*F* = 3.39, *p* < .005), while the differences were nonsignificant among water depth and nutrients (*F* = 2.10, *p* = .060), between species and nutrients (*F* = 2.35, *p* = .036). Afterward, RSR was also noted nonsignificant (*F* = 0.68, *p* = .086) in the interaction among water depth, nutrients, and species (Table 1). The values of RSR were decreased with increasing water depth in case of native *W. chinensis* under mixed culture as well as in monoculture. While, in case of *W. trilobata*, the RSR was sustained at 5S and 5F with N1, N2, and N3, followed by at 15F fluctuated water depth with N1 and N2 nutrient levels, respectively, under both mono and mixed culture (Figure 2).

**Figure 3:**
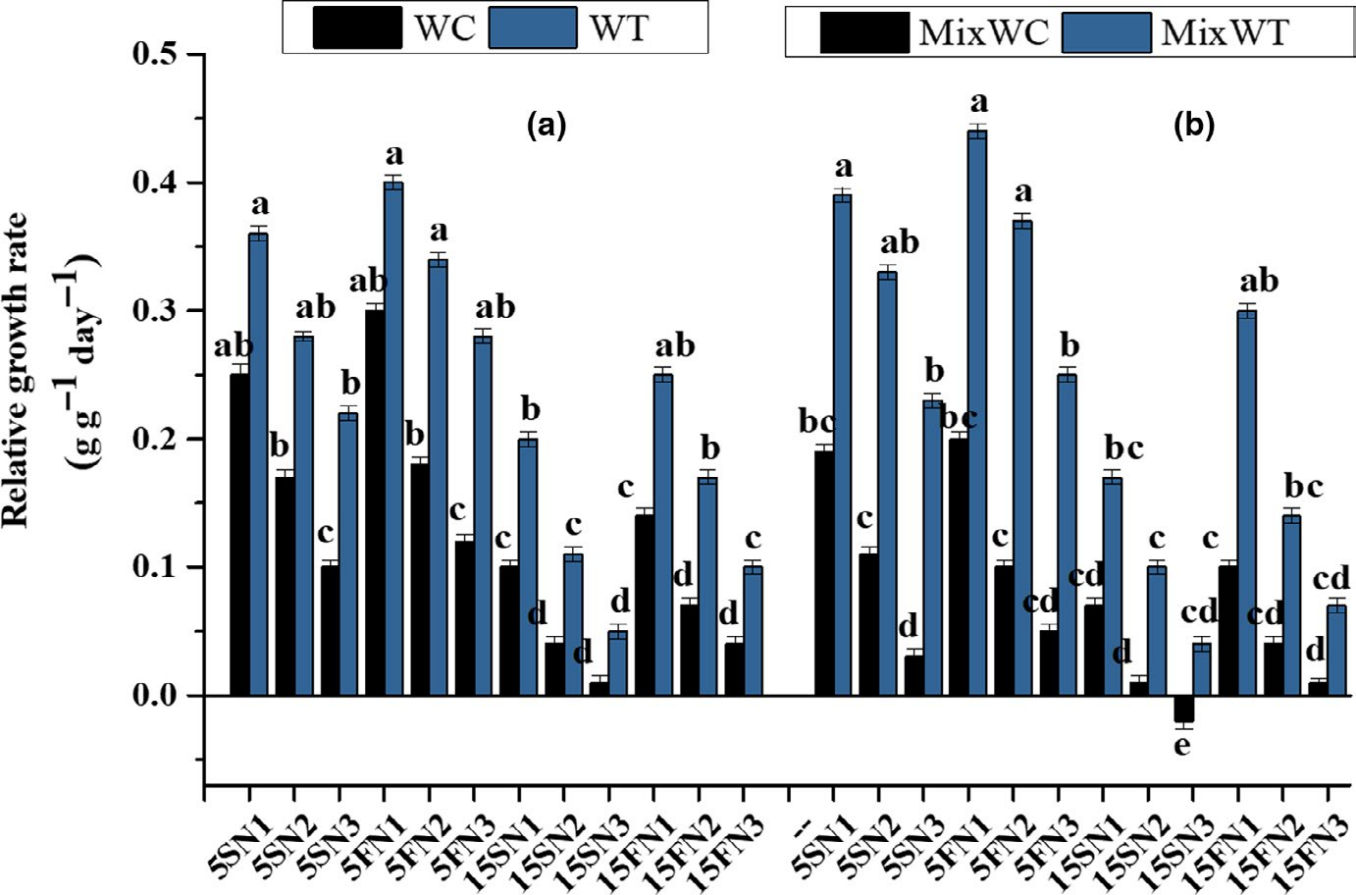
Effects of water depth fluctuations with different nutrient treatments on the (a) relative growth rate in monoculture, (b) relative growth rate in mixed culture of *W. trilobata and W. chinensis*. Mean + SE with different letters indicate a significant difference among mono and mixed culture treatments (at *p* < .05)

The effect of nutrients and water depth fluctuations of both the species appeared as necrosis and lost in their RGR at high water depth of 15S with low nutrient concentration of N3 (Figure 3). In case of *W. trilobata*, RGR was noted high at 5S and 5F water depth with N1, N2, and N3 nutrients concentration under both mono and mixed culture. Consequently, *W. trilobata* also showed survival and high RGR at 15S and 15F water depth fluctuations with N1 and N2 nutrient concentrations. Under low nutrient concentrations N3, the RGR of *W. trilobata* also decreased under mixed culture (Figure 3). Based on ANOVA (Table 1), RGR displayed significant values for interaction between water depth and nutrients (*F* = 34.58, *p* < .001), water depth and species (*F* = 194.06, *p* < .001), and between nutrients and species (*F* = 47.00, *p* < .001).

### 4.2 Physiological traits

**Figure 4:**
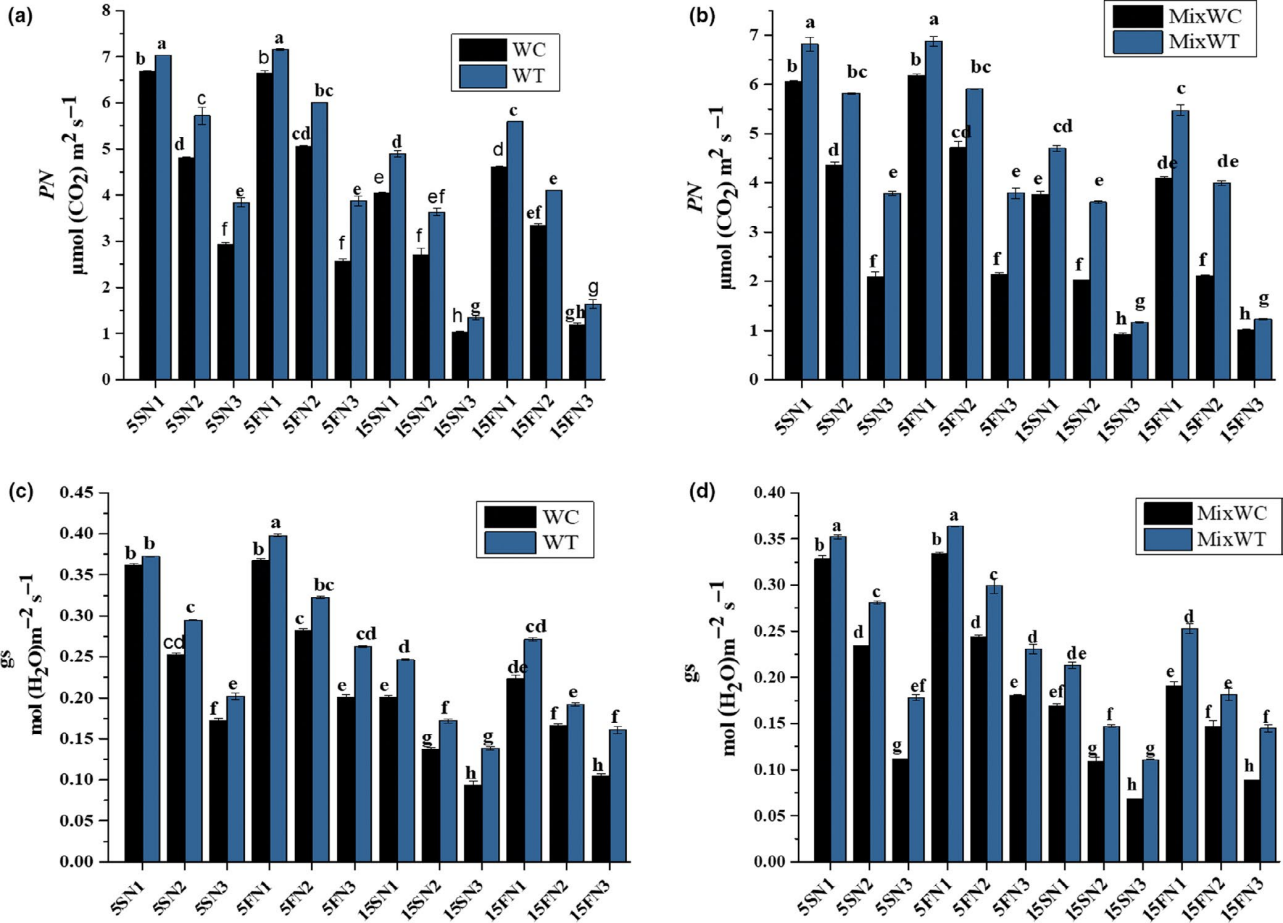
Effects of water depth fluctuations with different nutrient treatments on the (a) net photosynthetic rate in monoculture, (b) net photosynthetic rate in mixed culture, (c) stomatal conductance in monoculture, (d) stomatal conductance in mixed culture of *W. trilobata and W. chinensis*. Mean + SE with different letters indicate a significant difference among mono and mixed culture treatments (at *p* < .05)

In mono and mixed culture, ANOVA showed significant differences among all the treatments, species and their interactions except for the results of gs (Table 2). Physiological responses including *PN* and gs were lower in native *W. chinensis* under both the mono and mixed culture than in its invasive congener *W. trilobata* (Table 2; Figure 4a and b). Generally, the *PN* of both *W. trilobata* and *W. chinensis was* decreased with increasing water depth and by lowering the nutrients concentrations. However, *PN* of *W. chinensis* was more affected at high water depth of 15S with very low level of nutrients N3 under both mono and mixed culture. Consequently, the highest values of *PN* 7.20 and 7.03 μmol (CO_2_) m^−2^ s^−1^ were recorded for *W. trilobata* in monoculture, and 6.88 and 6.81 μmol (CO_2_) m^−2^ s^−1^ at 5FN1 and 5SN1 were noted in mixed culture, respectively, than in its competitor *W. chinensis* at same treatment levels (Figure 4a and b).

**Table 2:** Three‐way ANOVA results with interactions among water depth, nutrients levels, and species for the effects of water depth fluctuations with different nutrients treatments on physiological traits data for *W. trilobata* and *W. chinensis*

Factors	Physiological characteristics			
	PN	Gs	Ci	Chl
	(μmol (CO_2_) m^−2^ s^−1^)	(mol (H_2_O) m^−2^ s^−1^)	μmol/mol	(SPAD)
**WD**	3,751.49**	6.82**	1,770.56**	3,826.50**
***N***	11,157.36**	5.43*	2,201.18**	5,514.84**
**S**	941.46**	1.96^ns^	272.63**	514.16**
**WD** * ***N***	13.269**	1.10^ns^	26.84**	113.15**
**WD** * **S**	5.55**	0.99^ns^	7.79**	26.91**
***N*** * **S**	19.24**	1.06^ns^	2.87^ns^	11.08**
**WD** * ***N*** * **S**	21.91**	1.03^ns^	3.55**	3.24**

Abbreviations: *N*, nutrient treatments; ns, nonsignificant; S, species; WD, water depth.

* Indicates significant values at *p* < .005.

** Indicates significant values at *p* < .001.

When considering the different concentration of nutrients and water depth fluctuations, the similar pattern was noted for gs as found in *PN* in both *W. trilobata* and *W. chinensis*. There were significance differences in gs among water depth (*F* = 6.82, *p* < .001), nutrients (*F* = 5.43, *p* < .001), but nonsignificant among species and their interaction (Table 2). Upon treatments, in case of *W. chinensis*, a substantial decrease in gs was observed at 15S static water depth with low concentration of nutrients N3 under both mono and mixed culture than in its invasive competitor *W. trilobata* (Figure 4c and d). On the other hand *W. trilobata* competed well with their native competitor *W. chinensis* and showed high values of gs from low to high water depth and nutrients concentration under both mono and mixed culture (Figure 4c and d).

**Figure 5:**
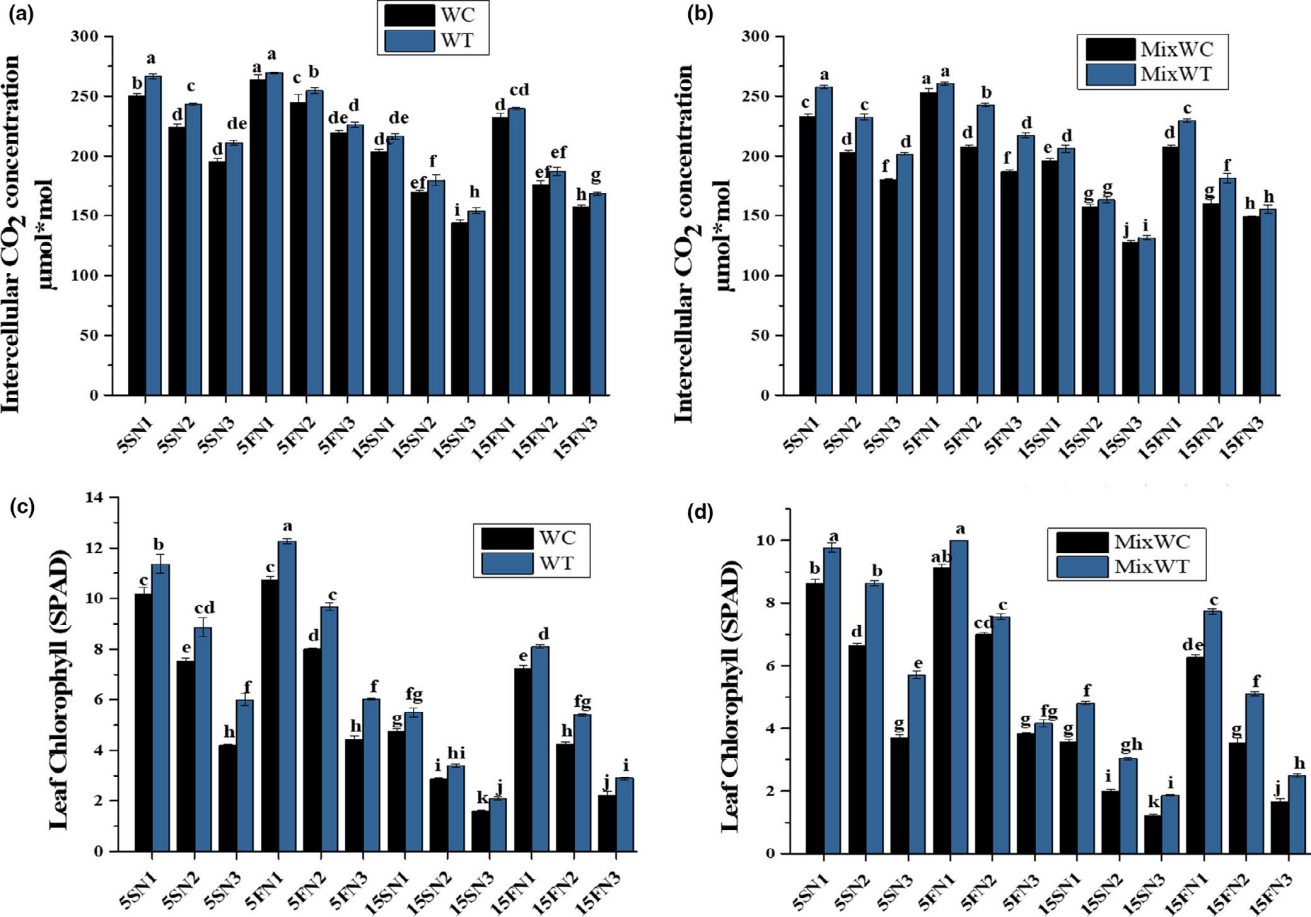
Effects of water depth fluctuations with different nutrient treatments on the (a) intercellular CO_2_ concentration in monoculture, (b) intercellular CO_2_ concentration in mixed culture, (c) leaf chlorophyll in monoculture, (d) leaf chlorophyll in mixed culture of *W. trilobata and W. chinensis*. Mean + SE with different letters indicate a significant difference among mono and mixed culture treatments (at *p* < .05)

The intercellular CO_2_ concentration (Ci) is another parameter that has been used to estimate the effects of hydrological variations on the physiology of *W. trilobata* and *W. chinensis*. The values of Ci became lower with increasing water depth and by lowering the nutrient concentrations (Figure 5a and b). Significant effect noted on Ci due to water depth (*F* = 1,770.56, *p* < .001), nutrients (*F* = 2,201.18, *p* < .001), and species (*F* = 272.63, *p* < .001). It was also recorded significant in their interaction as water depth and nutrients (WD × N), water depth and species (WD × S), nutrients and species (N × S), and water depth, nutrients and species (WD × N × S) (Table 2). Ci was found significantly higher in *W. trilobata* than *W. chinensis* under both mono and mixed culture (Figure 5a and b).

Leaf Chl is another physiological parameter which is affected by nutrients concentrations and water depth variations. Based on the ANOVA results, the values of Chl were noted significant in water depth (*F* = 3,826.50, *p* < .001), nutrients (*F* = 5,514.84, *p* < .001), and species (*F* = 514.16, *p* < .001). It was also recorded significant in their interaction as water depth and nutrients (WD × N), water depth and species (WD × S), nutrients and species (N × S), and water depth, nutrients and species (WD × N × S) (Table 2). *W. trilobata* competed well with their native competitor *W. chinensis* and showed higher values of Chl from low to high water depth with different nutrient concentrations, that is, at N1, N2, and N3 under both mono and mixed culture (Figure 5c and d).

### 4.3 Competitive interaction index

**Figure 6:**
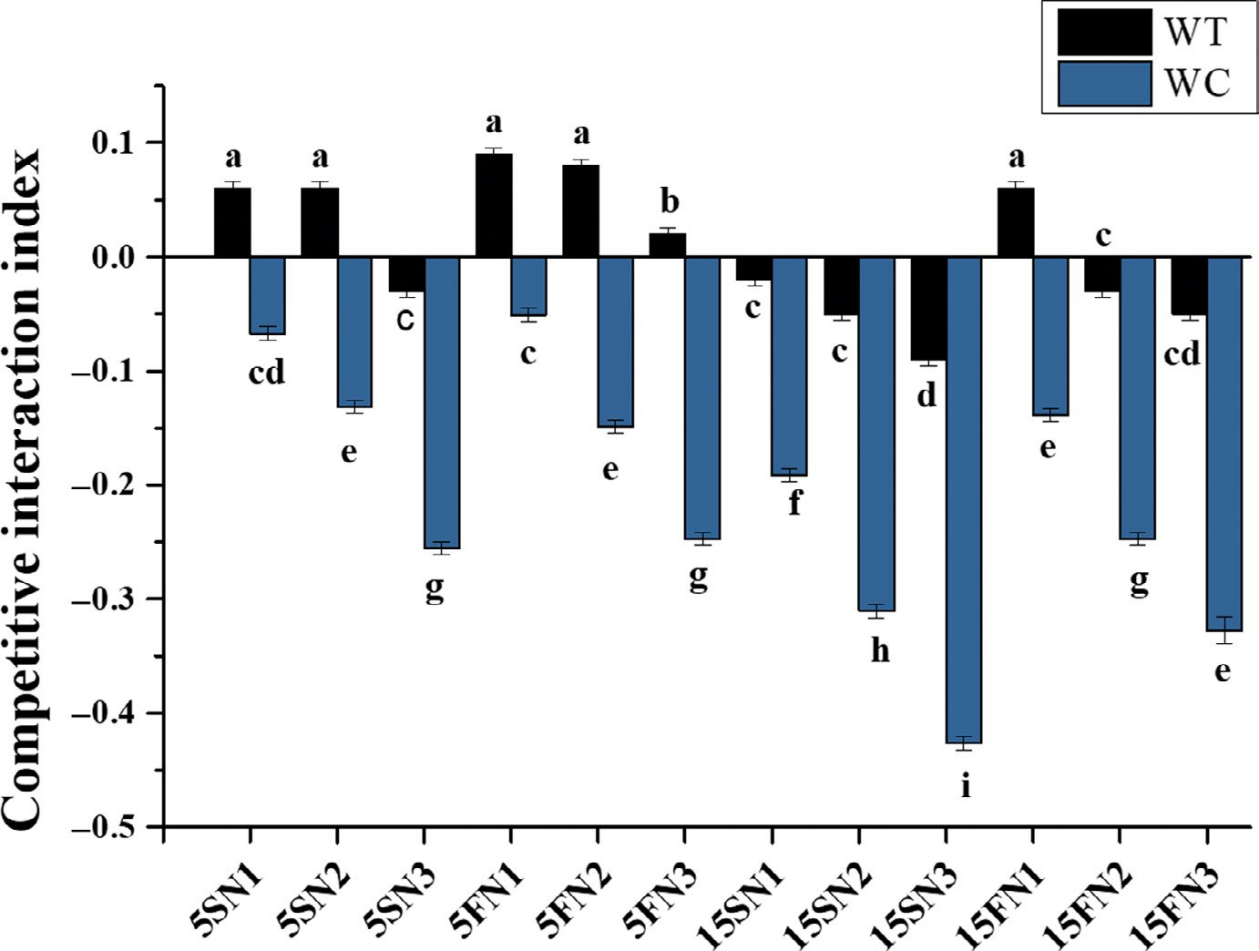
Effects of water depth fluctuations with different nutrient treatments on the competitive interaction among *W. trilobata and W. chinensis*. Mean + SE with different letters indicate a significant difference among mono and mixed culture treatments (at *p* < .05)

The values of CII of *W. trilobata* were higher and positive than CII values of *W. chinensis* (Figure 6). The average values of CII for *W. trilobata* were positive when grown at 5 cm water depth with low (N3) and high (N1) nutrient concentrations either the condition was static or fluctuated and were negative at the 15 cm water depth with low and high nutrient concentrations except for the treatment of 15FN1 (Table 3; Figure 6)., The mean values for *W. chinensis* were decreased with increasing water depth from 5 to 15 cm under both the conditions, that is, static or fluctuated; the values in all treatments were noted negative. The value at 15SN3 treatment was noted more negative, showed the sensitivity of *W. chinensis* in mixed planting than its competitor *W. trilobata* (Figure 6). However, the effect of water depth and nutrients on CII was noted significant. The differences were significant in CII values for both the species among the water depth (*F* = 0.132, *p* < .001), nutrients (*F* = 0.136, *p* < .001), species (*F* = 1.003, *p* < .001), and their interaction (*p* < .001) except the interaction between water depth and nutrients which was nonsignificant (*p* = 0.168) (Table 3).

**Table 3:** Three‐way ANOVA results with interactions among water depth, nutrients levels, and species for the effects of water depth fluctuations with different nutrients treatments on competitive interaction index data for *W. trilobata* and *W. chinensis*

Factors	Competitive interaction index (CII)		
	SS	F	P
WD	0.397	0.132	<0.001
*N*	0.272	0.136	<0.001
S	1.003	1.003	<0.001
WD* *N*	0.103	0.017	0.168
WD*S	0.094	0.031	<0.005
*N* *S	0.055	0.028	<0.005
WD* *N* *S	0.098	0.016	. <0.005

Abbreviationd: *N*, nutrient treatments; ns, nonsignificant; S, species; SS, sum of squares; WD, water depth.

* indicates significant values at *p* < .005.

** indicates significant values at *p* < .001.

## 5 DISCUSSION

The growth of *W. trilobata* and *W. chinensis* was significantly affected by hydrological variations either by increasing the water depth or by varying the nutrient concentrations (Table 1 and 2). The RGR of *W. chinensis* was decreased in mono and in mixed culture at 15S and 15F water depths with N1, N2, and N3 nutrient concentrations (Figure 4) because the 15 cm depth of water was high than the initial height of both *W. trilobata* and *W. chinensis*. Oxygen supply and carbohydrate use efficiency may reduce in this condition and in return nutrient consumption and an aerobic respiration increase. It led to an overall reduction in TFW and TDBm of both the species. Normally, under stress, the mother ramets provide nutrients to the water‐stressed and nutrient‐deficient seedlings through clonal integration (Gibbs & Greenway, 2003). However, in our study, at start of the experiment, we took single ramets for all plants of both the species, and the preliminary height was lower than the static‐water level of 15 cm and at an equal level of 5 cm. That is the reason that *W. chinensis* lost more biomass as shown by the dry weight of shoot and root in the 15 cm treatments than in the 5 cm treatments. We predicted that the 15 cm water level will be good only for *W. chinensis* when the nutrients level is high. While, *W. trilobata* survived and competed well under mono and mixed culture at 5 cm water depth as well as in 15 cm water depth with N1, N2, and N3 nutrient concentrations (Figures 1 and 2) due to its high phenotypic plasticity. Many invasive species have an ability to survive due to their morphological adaptation to submergence stress through their elongating of shoots and roots (Wang, Chen, Liu, & Li, 2014). Previous studies specify that *W. trilobata* spreads rapidly through the elongation of clonal ramets (Dai, Fu, Qi, et al., 2016a; Qi et al., 2014; Si et al., 2014), and their fast dispersion through vegetative propagation is one of the fundamental factors for the survival and successful invasion of *W. trilobata* in worst conditions (Song et al., 2010; Wu, Hu, & Chen, 2005).

Hydrological fluctuations cause plants to suffer from oxygen deficiency, resultantly acetaldehyde is produced and this damages the plant growth (Luo et al., 2012; Steffens, Steffen‐Heins, & Sauter, 2013). However, the hydrological fluctuations such as water depth variation and different nutrient concentrations have great effect on the growth of the studied invasive species in mono and mixed culture and its native congener *W. chinensis* (Table 1 and 2). In this, the fluctuation up to 5 cm range was low to affect the growth rate of *W. trilobata* and *W. chinensis*. Additional possible description for this effect may be that both species have capability to tolerate such fluctuations of water depth and show the compensatory growth. In some studies, it has been found that some wetland plants can stand against fluctuations up to 30 cm water depth without significant loss in the biomass (Wang, Zhang, et al., 2016a; Zhou et al., 2018). Luo, Jiang, Li, and Yu (2015) demonstrated that small variation in hydrological condition did not affect the plant's growth in wetland.

With the water depth fluctuation, there are other sources of stress that could affect the growth of target plants, such as nitrogen pulses, changed water content and low nutrients (Sun, Ding, & Ren, 2009; Wang et al., 2015). Zhang et al. (2016) suggested that increasing concentration of nutrients promoted growth of invasive *Alternanthera philoxeroides* and alleviated the stress of submergence. In our study, the reduction in the RSR indicated that increased water depth with low nutrients availability inhibited the root growth of the *W. chinensis* (Figure 2). However, the change in water depth at 5 cm did not affect the RSR and RGR under any nutrient conditions except N3, which suggested that nutrient levels also had an impact on growth of the native *W. chinensis* even at small water depth variations (Figures 2 and 3). High nutrient concentrations produced more biomass in *W. trilobata* allocated to shoot and root to adapt the water depth fluctuation from low to high levels, agreeing by the statement of Zhang et al. (2016) that more biomass allocated to stem alleviating the negative effects of submergence in invasive *Alternanthera philoxeroides*. In fluctuated water depth and nutrient deficit conditions, *W. trilobata* increased shoot biomass, which could increase the O_2_, CO_2,_ and light uptake, and promote its growth. Compared to *W. trilobata*, *W. chinensis* displayed a relatively higher reduction in growth traits in mixed culture, signifying higher sensitivity of it to increased water depth with nutrient deficiency conditions. Thus, high reduction in the growth traits and higher consumption of carbohydrates during limited available nutrients in high water depth might decrease the tolerance of *W. chinensis* and make it sensitive.

Hydrological fluctuation are also has a great impact on other factors like interspecific and intraspecific interactions in the wetland ecosystem (Wang, Shi, et al., 2016b). During the interaction, there is a trade‐off among plants between facilitation and competition, which means that in a suitable environment, plants show better competition and in a stressful environment exhibit more facilitation (Zhou et al., 2017). Fluctuation of water depth with nutrient treatments significantly affected the competitive interaction index between *W. trilobata* and *W. chinensis* (Table 3; Figure 6). *W. trilobata* benefited more from the competitive interaction than *W. chinensis*. *W. trilobata* performed better in mixed culture because of its survival and competitive ability even under high fluctuating water depth conditions at 15 cm with low‐nutrient treatments than *W. chinensis*. Here, *W. chinensis* might be suppressed by two factors; one is competition with *W. trilobata* and other is its sensitivity to water depth variations with nutrient deficiency. This outcome mainly happened because every plant species has different tolerance levels to worse environmental conditions, and here *W. trilobata* appears to be better capable to acclimate to greater stress by fluctuation in water depths and decreased nutrients. In addition, Deegan, White, and Ganf (2007) established treatments of hydrological fluctuation for wetland species; *Typha domingensis* responded depressingly to increasing water depth while, *Triglochin procerum* did not exhibit any response to increasing amplitude because of its better competitive ability.

*W. trilobata* and *W. chinensis* also responded differently in their physiological traits. *W. trilobata* generally exhibited their better ability to maintain photosynthesis, gs, Ci, and Chl to accumulate carbohydrates than *W. chinensis* under both mono and mixed cultured (Table 2, Figures 4 and 5). The ability of leaves of *W. trilobata* to sustain their photosynthetic rate in high water depth and nutrient deficit conditions could allow rapid carbon gain and the production part of photosynthesis might have been used for the regeneration of ribulose‐1,5‐disphosphate to promote the growth. These results agree with the preceding findings that invasive plants have significantly higher values of photosynthetic traits than native plants (Godoy et al., 2011; Kleunen, Weber, et al., 2010b). Similarly, Chen et al. (2013) found that invasive plants have high growth rate than native plants because of its better photosynthetic ability, and (Geng et al., 2006) noticed that invasive plants can showed higher competitive ability in both low water and waterlogged conditions. Therefore, flexibility, more competitive ability and high photosynthetic capacity may contribute to the invasiveness of *W. trilobata* under high water depth with high nutrient concentration in wetland ecosystem.

## 6 CONCLUSIONS

Invasive *W. trilobata* exhibited an escape strategy in order to adjust in high water depth with nutrient poor environment. While, *W. chinensis* presented lower values of physiological traits, with greater loss in the biomass. *W. chinensis* showed sensitivity because of its lower plasticity and poor competitive ability to water depth at 15 cm with all levels of nutrients except N1 under both mono and mixed culture. Higher tolerance of *W. trilobata* to water depth at 15 cm even with low nutrients N3 exhibited its higher photosynthetic capacity. High nutrients at N1 promoted the growth of *W. trilobata* and led it to its survival under high water depth, which may partially describe the ability of *W. trilobata* to invade waterlogged habitats in wetland. Moreover, these consequences may also contribute to our understanding about wetland invasive communities that how invasive species respond to the natural fluctuations and hydrological conditions modified by humans in the wetland. However, further studies based on growth and physiological traits will be needed to evaluate the invasions of invasive species at wetland in response to multiple environmental stresses.

## CONFLICT OF INTEREST

We have no conflicts of interest to declare.

## AUTHOR CONTRIBUTION

For this research articles, the individual contributions is listed as “conceptualization, Q. J. and S. F.; methodology, Q. J.; software, Q. J.; validation, Q. J., and A. A.; formal analysis, A. A. and I. U; investigation, S. F.; resources, D. D.; data curation, M. S. U and R. K.; writing—original draft preparation, Q. J.; writing—review and editing, S.F and A. A.; visualization, S. F.; supervision and funding acquisition, D. D.

## Data Availability

The data associated with this publication are deposited at Dryad data repository. Data files title: Fluctuated water depth with high nutrient concentrations promote the invasiveness of Wedelia trilobata in wetland submitted with https://doi.org/10.5061/dryad.9w0vt4bb1
